# Supplementary material for: A Metal Hydride Compressor Concept using Hydrogen as a Heat Transfer Fluid
Source: Commun Eng. 2026 Feb 20;5:49. doi: 10.1038/s44172-026-00615-6 (PMC12988172; doi:10.1038/s44172-026-00615-6)
Supplement: Supplementary file 2 — Supporting material [file 44172_2026_615_MOESM2_ESM.pdf]

Supplementary Information

A Metal Hydride Compressor Concept using Hydrogen as a Heat Transfer Fluid

Lukas Fleming[1,2], Maximilian Passing[1,2], Julián Puszkiel[1,2,*], Thomas Klassen[1,2], Julian Jepsen[1,2]

1 Faculty of Mechanical and Civil Engineering, Helmut-Schmidt-University, Hamburg, Germany
2 Institute of Hydrogen Technology, Helmholtz-Zentrum Hereon, Geesthacht, Germany
* julian.puszkiel@hereon.de

Contents

Supplementary Notes 1: Additional Information on the System Simulation 1
Supplementary Notes 2: 1D Metal Hydride Bed Model 3
Supplementary Notes 3: Sensitivity Analysis 4
Supplementary Notes 4: Verification by FEM Model 10
Supplementary Notes 5: Heat Transfer Coefficient 14
Supplementary Notes 6: BET area measurement (surface area): Results 15
References 17

Supplementary Notes 1: Additional Information on the System Simulation

For a better understanding of the system behavior, Supplementary Fig. 1 illustrates the hydrogen inlet and outlet flows, as well as the hydrogen saturation levels of both tanks. While tank I is connected to the hot loop and therefore desorbed, tank II is connected to the cold loop and absorbed. In accordance with this, a hydrogen flow into the compressor system and an outlet flow leaving the compressor system occur simultaneously. When both reactions come to an end, the connection between the tanks and loops is switched over, indicated by the vertical lines. As described in the main text, the tanks are first isolated from the two loops and then equilibrated in terms of gas phase pressure. This leads to the extra spikes that can be observed during the switch over phase. Tank II is absorbing an additional amount due to the high-pressure gas phase in the just desorbed tank I. The opposite process occurs in tank I, as the pressure drops below the former equilibrium. When the tanks are then reconnected back to the respective other loop, those additional capacities can be desorbed and, respectively, absorbed additionally. That way, hydrogen capacity is "transferred" from one tank to the other and is not entirely lost by being re-absorbed inside the same tank. This helps eliminate the negative impact of “dead volume” on productivity.

The isolation of the tanks from the loops during switch-over also prevents pressure equalization between the loops. As a result, the gas-phase volumes of the outer loops (dead volume) have no significant influence on the achievable productivity.

Supplementary Fig. 2 provides an overview of the simulation setup in Aspen Custom Modeler®.

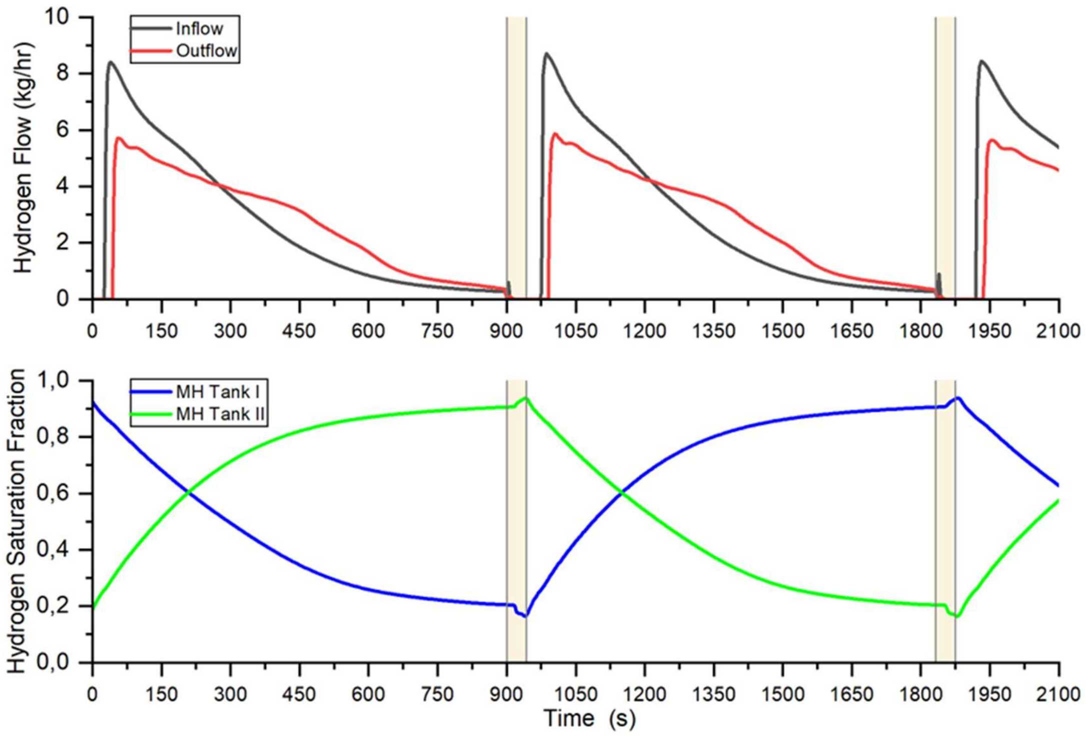

Supplementary Fig. 1: *The upper graph shows the inlet and outlet flow of the compressor system, caused by the absorption and desorption processes in the MH tanks, shown in the lower graph. The vertical lines indicate the switch-over process by valves V1-V8. The data is taken from the simulation run 3 of the HP sensitivity analysis (Supplementary Supplementary Table 2)*

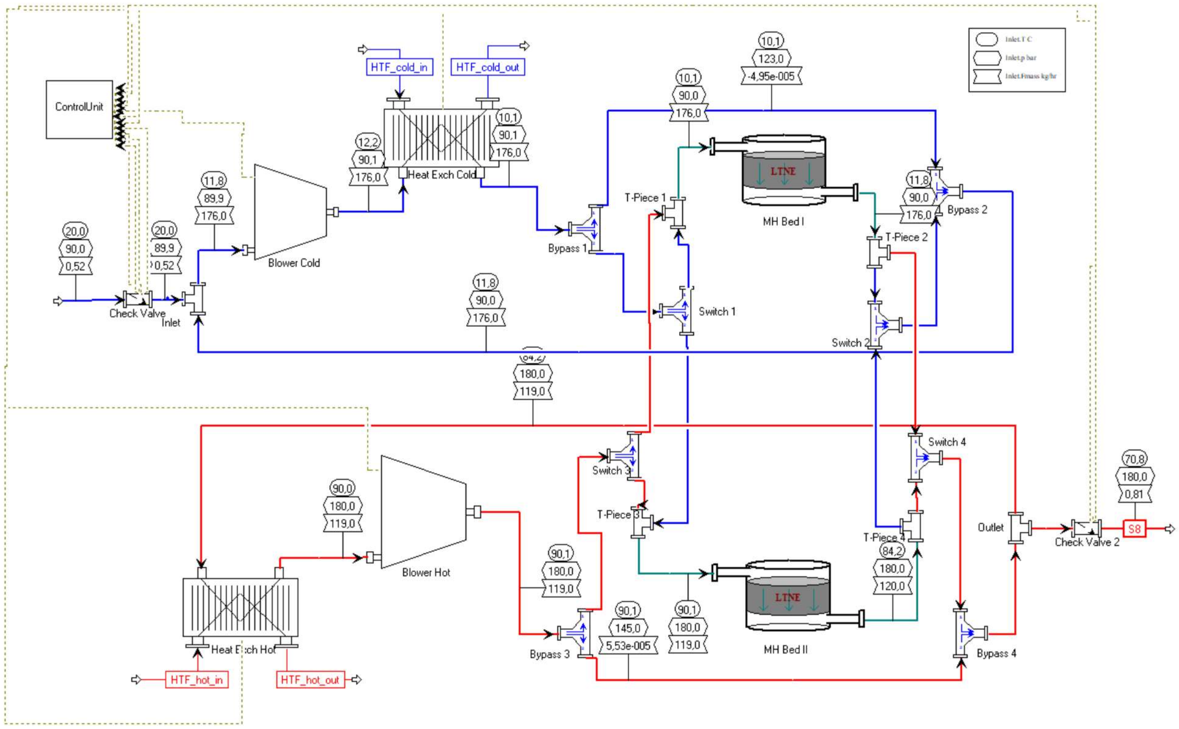

Supplementary Fig. 2: *Simulation setup of the Hydrogen Loop compressor system in Aspen Custom Modeler®. The 1D metal hydride bed models representing the processes inside the MH tanks are named "MH Bed I / II".Supplementary Table 2: Sample matrix and simulation results for the HP stage*

Supplementary Notes 2: 1D Metal Hydride Bed Model

Figure 1 in the main article illustrates that the MH material is arranged in multiple disc-shaped beds stacked vertically inside the tank. These beds are separated by flow gaps that allow hydrogen to enter each disc and exit the tank after passing through the material. In the ACM sub-model, this geometry is simplified by representing the stacked discs as a single equivalent disc with the same height *L* and an adjusted diameter to match the total mass of hydride in the tank. Previous FEM analysis has shown that transport processes in the bed are predominantly vertical, confirming that a 1D spatial resolution is sufficient. The gas-phase volume above and below the bed (i.e., the sum of all inter-disc flow gaps) is modeled as a lumped control volume.

Supplementary Fig. 3 illustrates the spatial discretization of the 1D bed model. For demonstration, the schematic refers to a single disc, whereas the implemented ACM model adjusts the diameter to represent the complete hydride inventory. The figure illustrates 6 nodes for clarity, whereas the simulations presented in this work utilize 10 nodes. Both hydride properties and hydrogen flow are resolved over these nodes. At each node, the following variables are computed using a first-order backward finite difference scheme:

- Rate of the hydrogen formation reaction, including equilibrium pressure and the kinetic equation
- Hydrogen saturation
- Heat transfer rate
- Temperature of the solid and the fluid
- Hydrogen mass stream, accounting for the amount of hydrogen absorbed or desorbed

The pressure drop is calculated for the bulk bed. It includes the change in porosity due to changes in crystalline density with hydrogen saturation. The resulting operative pressure is distributed equally over all nodes.

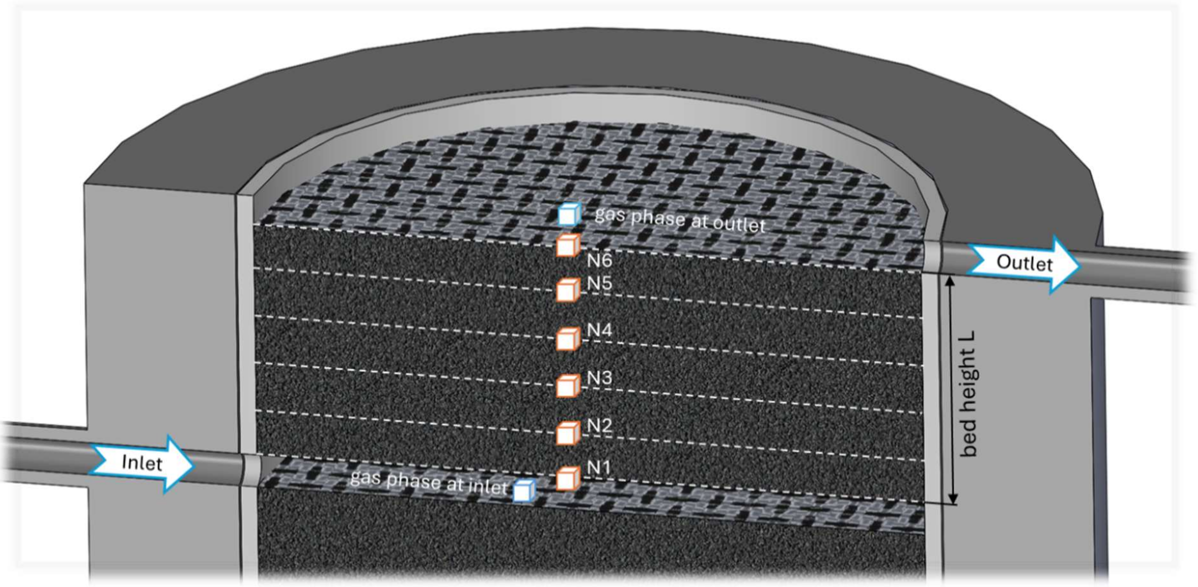

Supplementary Fig. 3: *MH tank sub-model. The illustration shows a setup with 6 nodes in the MH bed and point mass gas volumes at the inlet and outlet.*

The number of 10 nodes chosen for the simulations is a compromise between the resolution of the bed height and computational effort. In Supplementary Fig. 4, the simulation results of an absorption reaction are compared between the 1D and FEM models. Additionally, the results of a 1D simulation with 100 nodes are shown. It can be observed that the curves for 100 nodes are nearly identical to those for 10 nodes, except for the end phase of the reaction,

where the curve for 100 nodes follows the FEM results more closely. When running the whole system simulation in comparison between 10 and 100 nodes, the deviation in results (productivity and CoP) was less than 1%, while the computation time was substantially improved. Therefore, the MH tank sub-models were simulated with 10 nodes.

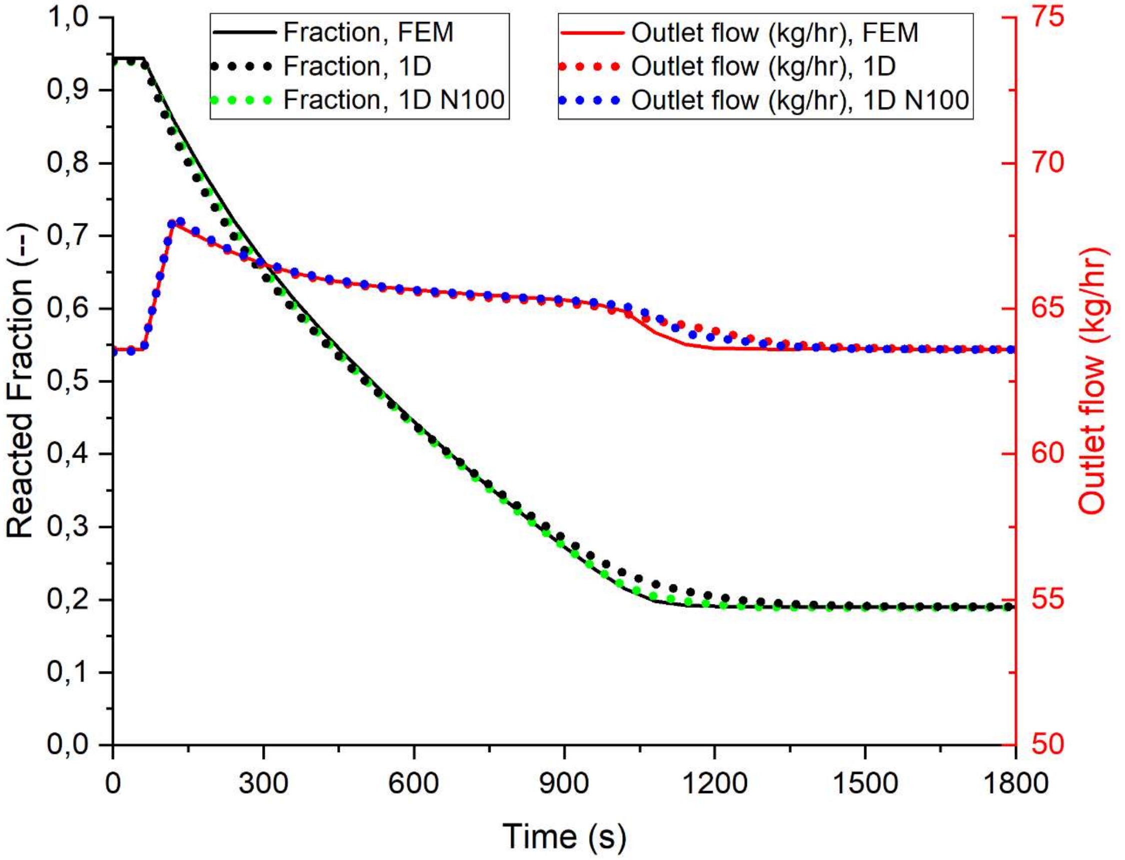

Supplementary Fig. 4: *Comparison of simulation results between the FEM-model, 1D-FDM-model with 10 nodes, and 1D-FDM-model with 100 nodes.*

Supplementary Notes 3: Sensitivity Analysis

In order to carry out a sensitivity analysis using the Morris method, value ranges are required for each parameter to be analyzed. Starting from a base case taken from the studies on productivity and efficiency, reasonable value ranges were selected. They can be found in the header of Supplementary Table 1. The table contains 7 columns for the parameters to be investigated, as well as 2 additional columns for the simulation results regarding the CoP (isothermal efficiency) and productivity. The 32 simulation runs in the parameter columns form the sample matrix that was generated according to the Morris method using the R package "sensitivity". All entries in the sample matrix and result vectors are color-coded by column to visualize the value distribution.

The choice of parameters and their value range for the sensitivity analysis is explained in more detail below:

- **P_1: Particle diameter.** From the analysis of activated and cycled metal hydride powders at the Hereon research center, a particle diameter of 20 µm is considered a representative value. It was assumed as the base value for the sensitivity analysis, and the range was set to be 80 % lower and higher, yielding 4 µm and 36 µm. This range

of particle sizes is typically represented in particle size distributions. For the analysis, a uniform particle size was chosen to show the direct effect of particle diameter on the efficiency and productivity.

- **P_2: Solid thermal conductivity.** One aim of this work is to demonstrate that the concept of using hydrogen gas convective heat transfer practically eliminates the influence of the effective thermal conductivity of the MH bed, unlike in conventional MH compressors. In the heat transfer equation for porous media (equation (2) in the main text), the conductive term includes $(1-\varepsilon)*k_s$, indicating that the effective thermal conductivity depends on the porosity ε and the solid thermal conductivity k_s. While the influence of the porosity is analyzed via the parameter P_4, the solid thermal conductivity remains to be investigated. The value range was set to be ± 50 % of a base value of 10.1 W/(m K) [1].
- **P_3: Height of the porous MH bed.** The height of the bed is included in the pressure drop calculation acc. to the Ergun equation. Moreover, it is interesting to see how high the individual beds inside the tank can be designed, reducing complexity with larger bed segments. Lastly, any effects coming from a potentially deviating temperature profile can be covered by varying the bed height.
- **P_4: Porosity of the MH bed.** Porosity is crucial in the pressure drop calculation. Along with porosity, the gas phase volume inside the tank has a significant effect on the performance of MH and mechanical compressors. Therefore, it is important to examine the extent to which porosity can be increased to minimize pressure drop while potentially compromising performance. The P&ID of the Hydrogen Loop compressor was designed to address the problem of dead space volumes inside the MH tanks, as shown here.
- **P_5: Efficiency of the hydrogen blower.** Through market research, an isentropic efficiency of roots blowers for hydrogen of approximately 40 % was found. The blower is a crucial component in the Hydrogen Loop compressor; therefore, any uncertainties in this regard are addressed by including the blower efficiency as a parameter and varying the base value by 25%.
- **P_6: Void volume of the cold and hot loop.** The piping, hydrogen blowers and heat exchangers come with significant internal gas phase volumes which, in an open system, would add to the dead space volume and reduce the compressor performance to practically zero. Any desorbed gas would increase the pressure in these volumes first, before leaving the compressor at a higher pressure level. Absorption would take the gas from the same volumes, effectively restricting the compression process to the inside of the compressor without any in- or output. The P&ID design of the Hydrogen Loop, however, aims not only to counteract the adverse effects of dead space inside the MH beds (P_4 and P_7) but also to address the void volumes of the two loops. This parameter is included to show the effectiveness of the P&ID design.
- **P_7: Height of the gaps between beds inside the MH tanks.** This parameter is included to vary the void volume inside the MH tank directly and to represent the engineering challenge of maintaining narrow hydrogen flow channels. It has a co-dependency with the bed height because the bed height determines the number of beds stacked to reach the total mass and hence the number of gaps.

Other parameters initially considered in the analysis are the surface area of the particles (a_{sf}) and the heat transfer coefficient (h_{sf}). Both form the interstitial convective heat transfer coefficient q_{sf} (equation (4) in the main text), which is part of the convective power term (the most right-hand term in equation (3) in the main text). In preliminary studies, the two parameters were each held constant and varied to observe their influence on heat transfer. It was apparent that their variations had little to no impact on the heat transfer and reaction process because their individual values are of such magnitude (1.46 $m^2\,g^{-1}$ and 16-

24 kW $m^{-2} K^{-1}$)) that q_{sf} remains at extremely high values and in consequence, the temperature difference (T_s – T_f) is equilibrated in a short period of time at every calculation node. This led to the conclusion that any uncertainties associated with the values for these two parameters have a negligible impact on the system and are therefore not included in the sensitivity analysis.

The Morris method was chosen for the sensitivity analysis. The background is best explained by [2] and is cited in the following paragraph:

The method of Morris allows to classify the inputs in three groups: inputs having negligible effects, inputs having large linear effects without interactions and inputs having large non-linear and/or interaction effects. The method consists in discretizing the input space for each variable, then performing a given number of OAT design. Such designs of experiments are randomly chosen in the input space, and the variation direction is also random. The repetition of these steps allows the estimation of elementary effects for each input. From these effects, sensitivity indices are derived.

Let us denote r the number of OAT designs (Saltelli et al. [78] propose to set parameter r between 4 and 10). Let us discretize the input space in a d-dimensional grid with n levels by input. Let us denote $E_j^{(i)}$ the elementary effect of the jth variable obtained at the ith repetition, defined as:

$$E_j^{(i)} = \frac{f(\mathbf{X}^{(i)} + \Delta e_j) - f(\mathbf{X}^{(i)})}{\Delta} \qquad (5.4)$$

where Δ is a predetermined multiple of $\frac{1}{(n-1)}$ and e_j a vector of the canonical base. Indices are obtained as follows:

- $\mu_j^* = \frac{1}{r}\sum_{i=1}^{r} |E_j^{(i)}|$ (mean of the absolute value of the elementary effects),
- $\sigma_j = \sqrt{\frac{1}{r}\sum_{i=1}^{r}\left(E_j^{(i)} - \frac{1}{r}\sum_{i=1}^{r} E_j^{(i)}\right)^2}$ (standard deviation of the elementary effects).

The interpretation of the indices is the following:

- μ_j^* is a measure of influence of the jth input on the output. The larger μ_j^* is, the more the jth input contributes to the dispersion of the output.
- σ_j is a measure of non-linear and/or interaction effects of the jth input. If σ_j is small, elementary effects have low variations on the support of the input. Thus the effect of a perturbation is the same all along the support, suggesting a linear relationship between the studied input and the output. On the other hand, the larger σ_j is, the less likely the linearity hypothesis is. Thus a variable with a large σ_j will be considered having non-linear effects, or being implied in an interaction with at least one other variable.

Then, a graph linking μ_j^* and σ_j allows to distinguish the three groups.

Morris method is applied on the flood example [Eqs. (5.2) and (5.3)] with $r = 5$ repetitions, which require $n = r(d+1) = 45$ model calls. Figure 5.2 plots results on the graph (μ_j^*, σ_j). This visualisation allows to make the following discussion:

- output S: inputs K_s, Z_v, Q, C_b et H_d are influent, while other inputs have no effects. In addition, the model output linearly depends on the inputs and there is no input interaction (because $\sigma_j \ll \mu_j^* \forall j$).

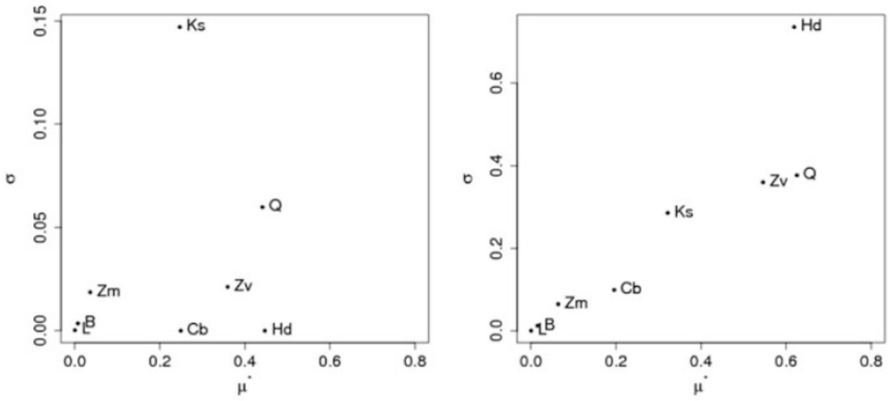

Fig. 5.2 Results of Morris method ($r = 5$ with 4 levels): outputs S (*left*) and C_p (*right*)

– output C_p: inputs H_d, Q, Z_v et K_s have strong influence with non-linear and/or interaction effects (because σ_j and μ_j^* have the same order of magnitude). C_b has an average influence while the other inputs have no influence.

Finally, after this screening phase, we have identified that three inputs (L, B and Z_m) have no influence on the two model outputs In the following, we fix these three inputs to their nominal values (which are the modes of their respective triangular distributions).

Further information can be found in [3] and [4].

Supplementary Table 1: *Sample matrix and simulation results for the LP stage*

in/outputs	P_1	P_2	P_3	P_4	P_5	P_6	P_7	y_1	y_2
variable	d_p0	ks_fix	l_bed	epsilon_0	eta_blower	V_outside	gap	CoP	product-ivity
unit	[m]	[W/m/K]	[m]	[--]	[--]	[m³]	[m]	[--]	[kg/hr]
nominal	0,00002	8	0,06	0,6	0,4	0,014	0,01		
variation	80%	50%	50%	25%	25%	80%	80%		
lower	4,0E-06	4	0,03	0,45	0,3	0,003	0,002		
upper	3,6E-05	12	0,09	0,75	0,6	0,025	0,018		
1	0,000036	6	0,09	0,675	0,375	0,02483	0,002	0,97	1,80
2	0,000036	12	0,09	0,675	0,375	0,02483	0,002	0,96	1,77
3	0,000036	12	0,09	0,675	0,375	0,00828	0,002	0,95	1,76
4	0,000036	12	0,09	0,675	0,375	0,00828	0,014	0,95	1,75
5	0,000012	12	0,09	0,675	0,375	0,00828	0,014	0,76	1,41
6	0,000012	12	0,09	0,675	0,6	0,00828	0,014	0,86	1,59
7	0,000012	12	0,045	0,675	0,6	0,00828	0,014	1,01	1,87
8	0,000012	12	0,045	0,45	0,6	0,00828	0,014	0,51	0,95
9	0,000004	12	0,03	0,525	0,3	0,00276	0,002	0,31	0,58
10	0,000004	6	0,03	0,525	0,3	0,00276	0,002	0,35	0,65
11	0,000004	6	0,03	0,75	0,3	0,00276	0,002	0,79	1,46
12	0,000004	6	0,03	0,75	0,3	0,00276	0,014	0,76	1,40
13	0,000004	6	0,03	0,75	0,525	0,00276	0,014	0,84	1,55
14	0,000028	6	0,03	0,75	0,525	0,00276	0,014	0,98	1,82
15	0,000028	6	0,075	0,75	0,525	0,00276	0,014	1,08	1,99
16	0,000028	6	0,075	0,75	0,525	0,01932	0,014	1,08	1,99
17	0,000004	4	0,075	0,75	0,3	0,00828	0,006	0,54	1,00
18	0,000004	10	0,075	0,75	0,3	0,00828	0,006	0,54	1,00
19	0,000028	10	0,075	0,75	0,3	0,00828	0,006	0,87	1,62
20	0,000028	10	0,075	0,75	0,525	0,00828	0,006	1,08	2,00
21	0,000028	10	0,075	0,525	0,525	0,00828	0,006	0,84	1,55
22	0,000028	10	0,075	0,525	0,525	0,00828	0,018	0,84	1,55
23	0,000028	10	0,075	0,525	0,525	0,02483	0,018	0,83	1,53
24	0,000028	10	0,03	0,525	0,525	0,02483	0,018	0,88	1,63
25	0,000036	4	0,075	0,675	0,3	0,00276	0,006	0,88	1,63
26	0,000036	4	0,03	0,675	0,3	0,00276	0,006	0,87	1,60
27	0,000036	4	0,03	0,45	0,3	0,00276	0,006	0,83	1,52
28	0,000036	4	0,03	0,45	0,3	0,01932	0,006	0,83	1,53
29	0,000036	4	0,03	0,45	0,525	0,01932	0,006	1,00	1,84
30	0,000012	4	0,03	0,45	0,525	0,01932	0,006	0,67	1,24
31	0,000012	4	0,03	0,45	0,525	0,01932	0,018	0,65	1,20
32	0,000012	10	0,03	0,45	0,525	0,01932	0,018	0,56	1,03

Supplementary Table 2: *Sample matrix and simulation results for the HP stage*

in/outputs	P_1	P_2	P_3	P_4	P_5	P_6	P_7	y_1	y_2
variable	d_p0	ks_fix	l_bed	epsilon_0	eta_blower	V_outside	gap	CoP	product-ivity
unit	[m]	[W/m/K]	[m]	[--]	[--]	[m³]	[m]	[--]	[kg/hr]
nominal	0,00002	8	0,06	0,6	0,4	0,014	0,01		
variation	80%	50%	50%	25%	25%	80%	80%		
lower	4,0E-06	4	0,03	0,45	0,3	0,003	0,002		
upper	3,6E-05	12	0,09	0,75	0,6	0,025	0,018		
1	0,000012	6	0,09	0,675	0,3	0,00828	0,006	1,20	1,87
2	0,000036	6	0,09	0,675	0,3	0,00828	0,006	1,45	2,25
3	0,000036	6	0,09	0,675	0,525	0,00828	0,006	1,64	2,55
4	0,000036	6	0,09	0,675	0,525	0,02483	0,006	1,65	2,56
5	0,000036	6	0,045	0,675	0,525	0,02483	0,006	1,61	2,51
6	0,000036	6	0,045	0,45	0,525	0,02483	0,006	1,48	2,31
7	0,000036	6	0,045	0,45	0,525	0,02483	0,018	1,47	2,28
8	0,000036	12	0,045	0,45	0,525	0,02483	0,018	1,38	2,15
9	0,000004	4	0,09	0,75	0,6	0,01932	0,006	1,18	1,84
10	0,000004	4	0,09	0,75	0,375	0,01932	0,006	1,04	1,61
11	0,000004	4	0,09	0,75	0,375	0,00276	0,006	1,08	1,69
12	0,000028	4	0,09	0,75	0,375	0,00276	0,006	1,59	2,47
13	0,000028	4	0,09	0,75	0,375	0,00276	0,018	1,50	2,34
14	0,000028	4	0,045	0,75	0,375	0,00276	0,018	1,42	2,21
15	0,000028	4	0,045	0,525	0,375	0,00276	0,018	1,21	1,88
16	0,000028	10	0,045	0,525	0,375	0,00276	0,018	1,27	1,98
17	0,000028	10	0,03	0,525	0,6	0,00828	0,002	1,56	2,42
18	0,000028	10	0,03	0,525	0,6	0,00828	0,014	1,52	2,36
19	0,000028	4	0,03	0,525	0,6	0,00828	0,014	1,60	2,48
20	0,000004	4	0,03	0,525	0,6	0,00828	0,014	0,76	1,18
21	0,000004	4	0,03	0,525	0,375	0,00828	0,014	0,66	1,04
22	0,000004	4	0,075	0,525	0,375	0,00828	0,014	0,33	0,51
23	0,000004	4	0,075	0,75	0,375	0,00828	0,014	1,13	1,76
24	0,000004	4	0,075	0,75	0,375	0,02483	0,014	1,12	1,74
25	0,000012	10	0,09	0,675	0,6	0,00276	0,018	1,47	2,29
26	0,000012	10	0,09	0,45	0,6	0,00276	0,018	0,53	0,83
27	0,000012	10	0,09	0,45	0,6	0,01932	0,018	0,54	0,84
28	0,000036	10	0,09	0,45	0,6	0,01932	0,018	1,22	1,90
29	0,000036	4	0,09	0,45	0,6	0,01932	0,018	1,22	1,91
30	0,000036	4	0,09	0,45	0,6	0,01932	0,006	1,23	1,91
31	0,000036	4	0,045	0,45	0,6	0,01932	0,006	1,58	2,46
32	0,000036	4	0,045	0,45	0,375	0,01932	0,006	1,30	2,03

The result of the analysis is given by a vector of the standard deviation of the elementary effects σ and the mean of the absolute value of the elementary effects μ*.

Supplementary *Table 3:* *Results of the Morris method for the LP and HP stage*

LP Stage				HP Stage			
	μ	μ*	σ		μ	μ*	σ
**p1**	0,33	0,33	0,13	**p1**	0,64	0,64	0,64
**p2**	-0,05	0,05	0,05	**p2**	-0,11	0,11	0,11
**p3**	-0,03	0,10	0,14	**p3**	-0,13	0,14	0,13
**p4**	-0,41	0,41	0,28	**p4**	0,33	0,45	0,73
**p5**	0,19	0,19	0,08	**p5**	0,25	0,25	0,21
**p6**	0,00	0,01	0,01	**p6**	-0,05	0,05	0,07
**p7**	-0,02	0,02	0,02	**p7**	-0,06	0,06	0,04

In the discussion, we refer to the impact of certain parameters and express it as a percentage. This is calculated by examining the effect on CoP and productivity when only that parameter is changed (one factor at a time). The following Supplementary Table 4 shows which simulation runs were used for comparison. They refer to the list of simulation runs in Supplementary Tables 1 and 2.

Supplementary Table 4: *Effect of P6 and P7 when varying one factor at a time*

**LP Setup**							
**Effect of the outer volumes, P6**				**Effect of the internal volume, P7**			
Sim. run	Parameter val	CoP result	Deviation	Sim. run	Parameter val	CoP result	Deviation
2	0,02483	0,95833312		3	0,002	0,95130365	
3	0,00828	0,95130365	**0,73%**	4	0,014	0,94920219	**0,22%**
15	0,00276	1,07690519		11	0,002	0,79265405	
16	0,01932	1,07727201	**-0,03%**	12	0,014	0,7560186	**4,62%**
22	0,00828	0,84050973		21	0,006	0,84037761	
23	0,02483	0,82978125	**1,28%**	22	0,018	0,84050973	**-0,02%**
27	0,00276	0,82583542		30	0,006	0,66948385	
28	0,01932	0,83283362	**-0,85%**	31	0,018	0,65174471	**2,65%**
**HP Setup**							
**Effect of the outer volumes, P6**				**Effect of the internal volume, P7**			
Sim. run	Parameter val	CoP result	Deviation	Sim. run	Parameter val	CoP result	Deviation
3	0,00828	1,64145903		6	0,006	1,48144577	
4	0,02483	1,64986073	**-0,51%**	7	0,018	1,46576469	**1,06%**
10	0,01932	1,03613553		12	0,006	1,59036037	
11	0,00276	1,08313514	**-4,54%**	13	0,018	1,50397702	**5,43%**
23	0,00828	1,13184861		17	0,002	1,56019562	
24	0,02483	1,11738043	**1,28%**	18	0,014	1,52326284	**2,37%**
26	0,00276	0,53396563		29	0,018	1,22335231	
27	0,01932	0,53737643	**-0,64%**	30	0,006	1,22744029	**-0,33%**

Supplementary Notes 4: Verification by FEM Model

The verification of the 1D model within the system simulation in Aspen Custom Modeler® (ACM) was performed using the finite element method (FEM) simulation software COMSOL Multiphysics®. The identical model equations were used as in the 1D system simulation. The geometry of a single metal hydride disc, with a height of 60 mm and a diameter of 651 mm,

was selected, featuring a free gas phase gap at both the inlet and outlet, with heights of 10 mm, respectively. The geometry was built as a 2D axisymmetric model. A mesh of triangles and rectangles was used at the wall interface to represent the fluid flow field. The skewness quality of the mesh showed a minimum value of 0.215 and an average quality of 0.847, with a total number of 38,940 elements and an element-to-volume ratio of 7.43E-4. The heat transport and fluid dynamic variables were solved using the PARDISO solver.

An absorption and desorption scenarios were simulated using ASM and COMSOL to compare and verify the validity of the 1D Model approach in system simulation. For absorption, an initial hydride fraction of 0.183 was set, leading to an initial pressure of 30 bar at a starting temperature of 90 °C. The desorption scenario began at a hydride fraction of 0.944, resulting in a pressure of 30 bar at an initial temperature of 10 °C. Starting from a homogeneous initial condition and after achieving a constant hydrogen flow field, the temperature of the incoming hydrogen flow is ramped within 10 s from 90 to 10 °C in the absorption case and from 10 to 90 °C in the desorption case. The fluid properties of the hydrogen were taken from the COMSOL materials database.

Heat transport in porous media with local thermal inequality

Analogue to the 1D model in ACM, a thermal local inequality between the solid (1) and fluid (3) phase was set, solving for each phase separately.

$$(1-\varepsilon)\rho_s c_{p,s}\frac{\partial T_s}{\partial t} + \nabla q_s = Q_{sf} + (1-\varepsilon)Q_s \quad (1)$$

ε is the porosity, ρ_s is the solid density, $c_{(p,s)}$ is the solid heat capacity, q_s is the heat conduction, Q_{sf} is the heat transfer between solid and fluid, and Q_s is the heat of the reaction. The solid heat conduction is given as (2):

$$q_s = (1-\varepsilon)\, k_s\, \nabla T_s \quad (2)$$

k_s is the solid thermal conductivity. The gas phase is calculated analogously to the solid phase (3):

$$\varepsilon\rho_f c_{p,f}\frac{\partial T_f}{\partial t} + \rho_f c_{p,f} u_f \nabla T_f + \nabla q_f = -Q_{sf} \quad (3)$$

ϱ_f is the gaseous density, $c_{(p,f)}$ is the gaseous heat capacity, u_f is the velocity of the gas, and q_f is the heat conduction of the gaseous phase.

For the heat exchange between the phases, the implemented interstitial convective heat transfer for spherical particles is used (4,5,6):

$$Q_{sf} = a_{sf}\, h_{sf}\, (T_s - T_f) \quad (4)$$

$$h_{sf} = \left[d_p \frac{1}{k_f Nu_{sf}} + \frac{1}{10 k_s}\right]^{-1} \quad (5)$$

$$Nu_{sf} = 2.0 + 1.1\, Pr^{1/3}\, Re \quad (6)$$

Nu_sf is the Nusselt number, Pr is the Prandtl number, Re is the Reynolds number, h_sf is the heat transfer coefficient, and a_{sf} is the specific surface area. The reaction heat Q_{sf} evolves within the porous media domain, depending on the reaction rate.

Free fluid flow and flow in porous media using the Brinkmann equation

The fluid dynamics were calculated for the free hydrogen flow and in the porous metal hydride bed. The hydrogen is set as a compressible fluid. The mass conservation for the compressible flow is given in (7).

$$\frac{\partial(\varepsilon\,\rho_f)}{\partial t} + \nabla\left(\rho_f u_f\right) = Q_m \quad (7)$$

The inertia term, the gravity force, as well as the turbulence were neglected. The flow model was selected to be valid in Darcy's range, as the prior flow analysis showed low velocities with dominant laminar flow.

$$\frac{\rho_f}{\varepsilon}\frac{\partial u_f}{\partial t} = \nabla\left(-pI + K\right) - \left(\frac{\mu}{\kappa} + \beta\rho_f\left|u_f\right| + \frac{Q_m}{\varepsilon^2}\right)u_f + F \quad (8)$$

$$K = \frac{\mu}{\varepsilon}\left(\nabla u_f + \left(\nabla u_f\right)^T\right) - \frac{2}{3}\frac{\mu}{\varepsilon}(\nabla u)I \quad (9)$$

$$F = -\rho_f\beta\left|u_f\right|u_f \quad (10)$$

p is the pressure, μ is the dynamic viscosity, κ is the permeability, Q_m is the mass source, and F is the Forchheimer drag term.

The simulation begins with no flow, which is then ramped up to a constant hydrogen flow of 63.6 kg/h in 1 s to establish a steady-state flow field. The outlet boundary condition is set to a continuous pressure of 30 bar.

Transport of diluted species

Within this module, the chemical reaction between the metallic and hydride phases is calculated using the kinetic model [5] and the thermodynamic model from [5,6]. The pressure and temperature state variables are taken from the modules mentioned above. The reaction is only calculated in the porous media domain.

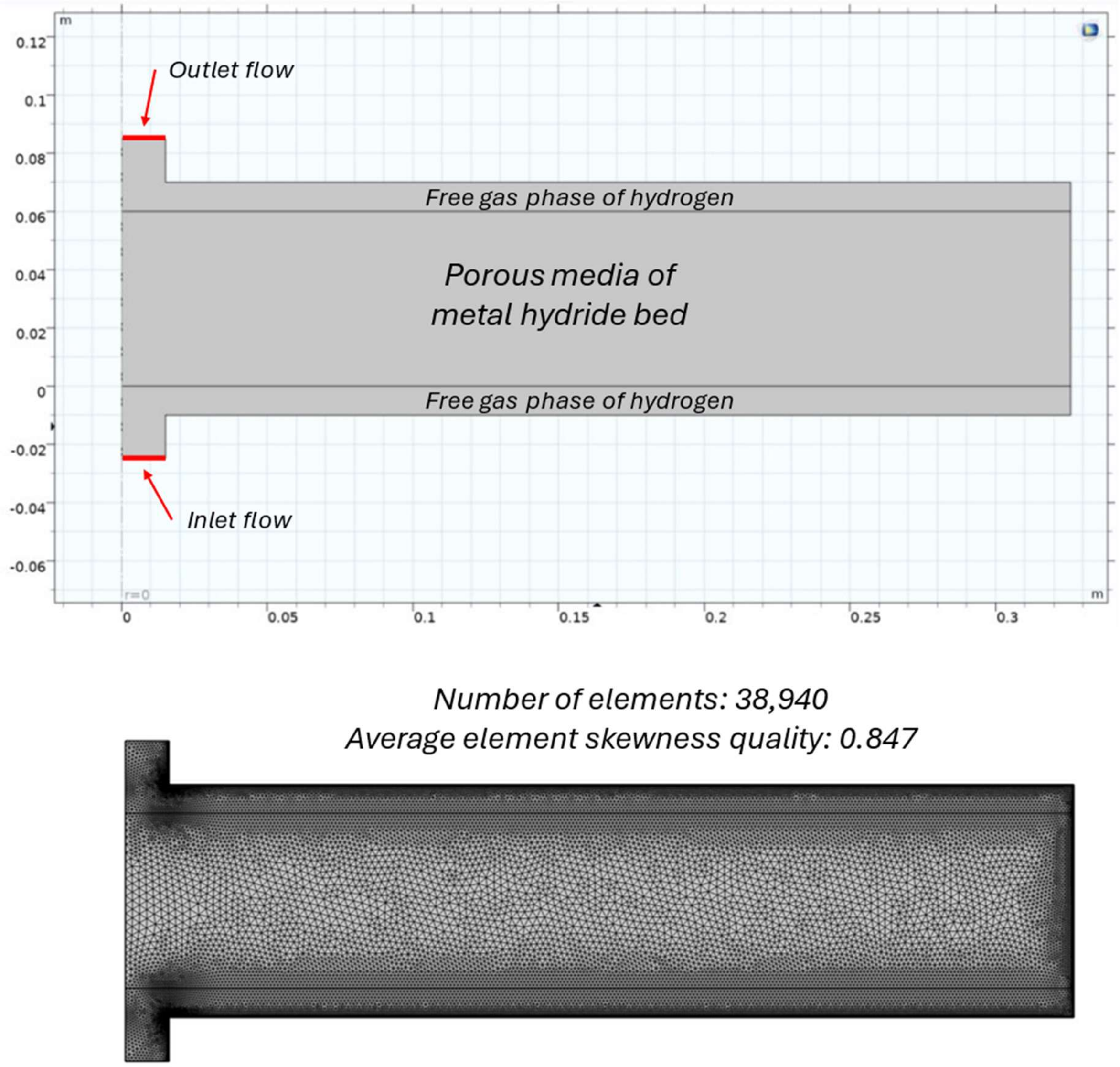

Supplementary Fig. 5: *Schematic of the FEM geometry, indicating the porous media and gas phase domains and the flow boundaries (left side) as well as the refining of the mesh of the given geometry (right side)*

Supplementary Fig. 6 presents a comparison of FEM and 1D-FDM for the absorption reaction in a single tank, supplementing the desorption results (Supplementary Fig. 4).

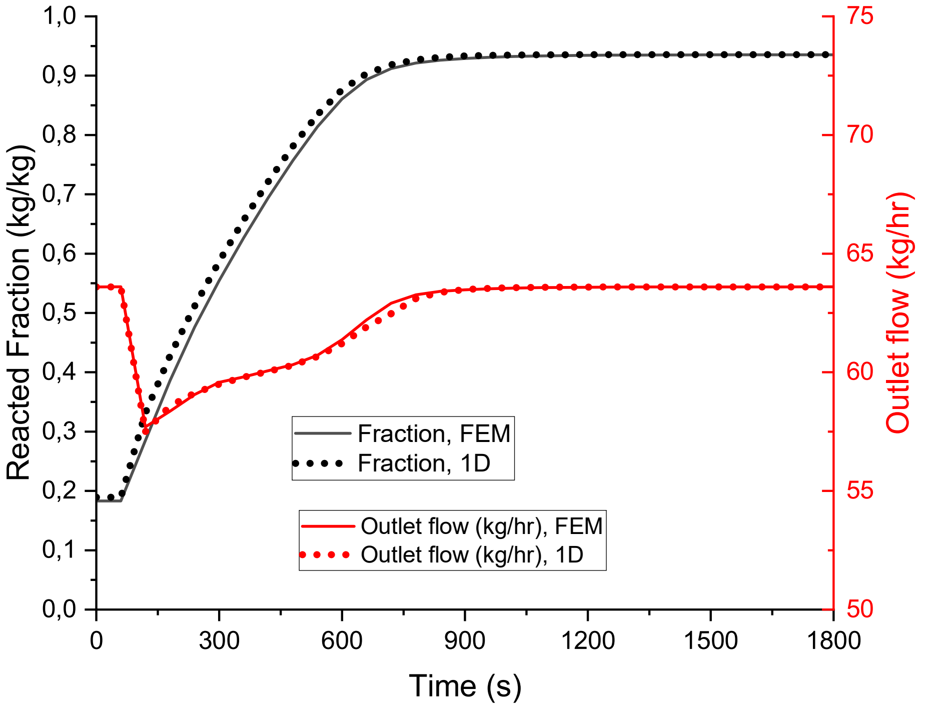

Supplementary Fig. 6: *Comparison of the absorption reaction. The 1D model is set up with 10 nodes.*

Supplementary Notes 5: Heat Transfer Coefficient

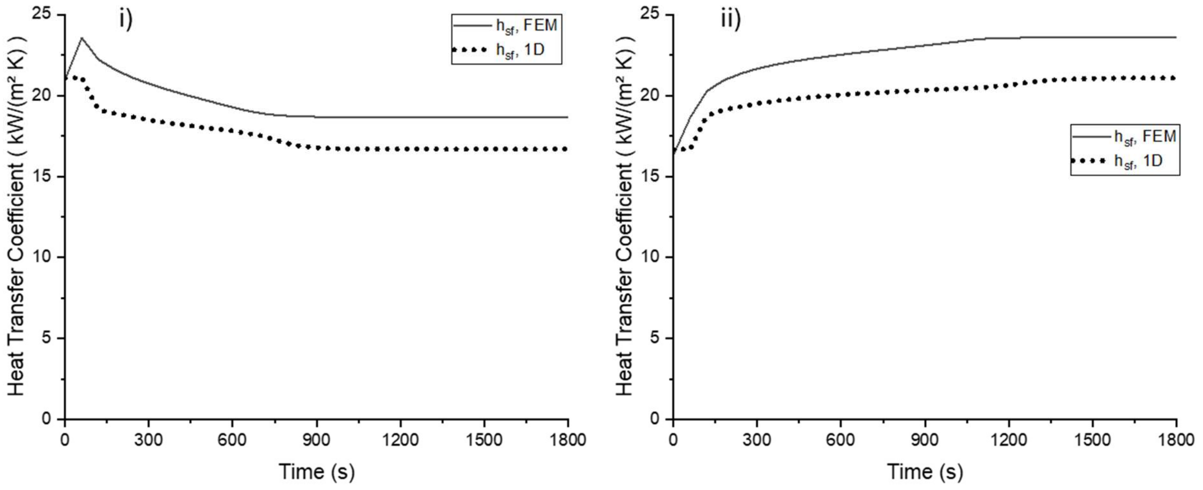

Supplementary Fig. 7: *Comparison of the calculated heat transfer coefficient between solid metal hydride particles and the hydrogen gas in the COMSOL Multiphysics FEM model and Aspen Custom Modeler 1D model, for i) absorption and ii) desorption.*

Supplementary Notes 6: BET area measurement (surface area): Results

Previous treatment: The hydride-forming alloy Hydralloy®C5 was activated to full capacity of 1.6-1.7 wt.% and cycled 5 times in a prototype tank containing 15 kg of material at 30 ºC under about 35 bar and 1 bar for absorption and desorption, respectively. Then, the material, after hydrogen interaction, was slowly oxidized in an air atmosphere for one week and then sieved to a particle size range of 20–30 microns. The oxidation does not alter the morphology and textural conditions of the particles, as observed by scanning electron microscope analysis of samples both with and without contact with air (photos and results not shown here). The sample for analysis was put into a 3/8-inch glass tube sample holder. The sample was degassed in the VapPrep unit (Micromeritics Instrument Corp.). The degassing procedure consisted of 48 hours of vacuum at room temperature, followed by ramping up to 400 ºC under vacuum and 24 hours at 400 ºC. After degassing, 3.7030 g of the powder sample was weighed in a 3/8-inch glass tube. Then, the sample was cooled down and connected to the TriStar□ II Plus surface area and porosity analyzer (Micromeritics Instrument Corp.).

Before starting the analysis, the sample was put under vacuum at room temperature for 5 hours to ensure the complete evacuation of air introduced during the connection to the device. The analysis used an adsorptive gas, nitrogen, in a liquid nitrogen bath (77.3 K). The equilibration interval for the BET area calculation was 10 seconds, and the minimum equilibration delay was at a relative pressure greater than or equal to 0.995 of 600 seconds. The mode used was low-area BET material in the relative pressure range (P/P_0) from 0 to 0.20.

The result shows that proper parameters were obtained based on the theory and physical meaning: C= 213.93 is in the range between 50 and 300, typical for most of the materials, the slope is positive (66.34328 ± 0.52756 g/mmol), and the Y-intercept is positive as well (0.31157 ± 0.05784 g/mmol). The C, slope, and Y-intercept verify that the device was working correctly (no leak) and that the selected pressure range was correct. The results are presented in Supplementary Fig. 8.

TriStar II Plus 3.04 | TriStar II Plus Version 3.04 | Serial # 3100 Unit 1 Port 2 | Page 4 of 19

Sample: Hydralloy_C5_Cycled_20-32microns
Operator: J.Puszkiel
Submitter:
File: C:\Users\5wds\Desktop\000-015.SMP

Started: 12.03.2025 10:22:52
Completed: 12.03.2025 12:28:07
Report time: 12.03.2025 12:28:07
Sample mass: 3,7030 g
Analysis free space: 28,1732 cm^3
Equilibration interval: 10 s
Sample density: 1,000 g/cm^3

Analysis adsorptive: N2
Analysis bath temp.: 77,300 K
Thermal correction: No
Ambient free space: 9,6493 cm^3 Measured
Port volume: -0,3169 cm^3
Low pressure dose: None
Automatic degas: No

Validation errors exist for this report. Review the validation report for details.

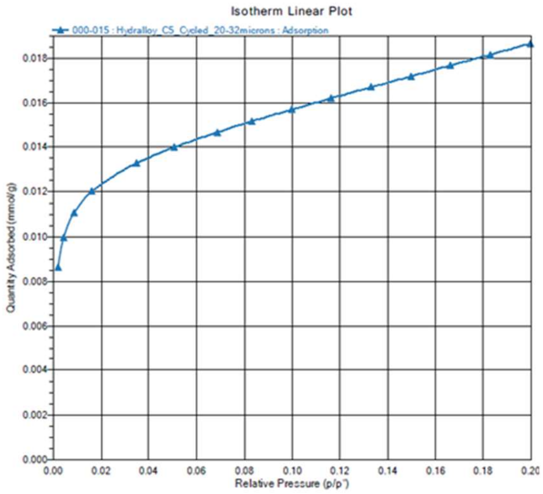

TriStar II Plus 3.04 | TriStar II Plus Version 3.04 | Serial # 3100 Unit 1 Port 2 | Page 5 of 19

Sample: Hydralloy_C5_Cycled_20-32microns
Operator: J.Puszkiel
Submitter:
File: C:\Users\5wds\Desktop\000-015.SMP

Started: 12.03.2025 10:22:52
Completed: 12.03.2025 12:28:07
Report time: 12.03.2025 12:28:07
Sample mass: 3,7030 g
Analysis free space: 28,1732 cm^3
Equilibration interval: 10 s
Sample density: 1,000 g/cm^3

Analysis adsorptive: N2
Analysis bath temp.: 77,300 K
Thermal correction: No
Ambient free space: 9,6493 cm^3 Measured
Port volume: -0,3169 cm^3
Low pressure dose: None
Automatic degas: No

Validation errors exist for this report. Review the validation report for details.

BET Report

BET surface area: 1,4636 ± 0,0117 m^2/g
Slope: 66,34328 ± 0,52756 g/mmol
Y-intercept: 0,31157 ± 0,05784 g/mmol
C: 213,932104
Qm: 0,01500 mmol/g
Correlation coefficient: 0,9995892
Molecular cross-sectional area: 0,1620 nm^2

Relative Pressure ($p/p°$)	Quantity Adsorbed (mmol/g)	$1/[Q(p°/p - 1)]$
0,002011377	0,00864	0,23314
0,004284408	0,00997	0,43150
0,008621524	0,01107	0,78572
0,016186912	0,01203	1,36753
0,034842710	0,01329	2,71602
0,050504515	0,01400	3,79846
0,068532271	0,01468	5,01334
0,083150671	0,01517	5,97963
0,099762326	0,01569	7,06392
0,116399545	0,01619	8,13465
0,133018579	0,01669	9,19383
0,149682314	0,01718	10,24831
0,166312265	0,01766	11,29487
0,182953575	0,01815	12,33706
0,199610364	0,01865	13,37492

TriStar II Plus 3.04 | TriStar II Plus Version 3.04 | Serial # 3100 Unit 1 Port 2 | Page 6 of 19

Sample: Hydralloy_C5_Cycled_20-32microns
Operator: J.Puszkiel
Submitter:
File: C:\Users\5wds\Desktop\000-015.SMP

Started: 12.03.2025 10:22:52
Completed: 12.03.2025 12:28:07
Report time: 12.03.2025 12:28:07
Sample mass: 3,7030 g
Analysis free space: 28,1732 cm^3
Equilibration interval: 10 s
Sample density: 1,000 g/cm^3

Analysis adsorptive: N2
Analysis bath temp.: 77,300 K
Thermal correction: No
Ambient free space: 9,6493 cm^3 Measured
Port volume: -0,3169 cm^3
Low pressure dose: None
Automatic degas: No

Validation errors exist for this report. Review the validation report for details.

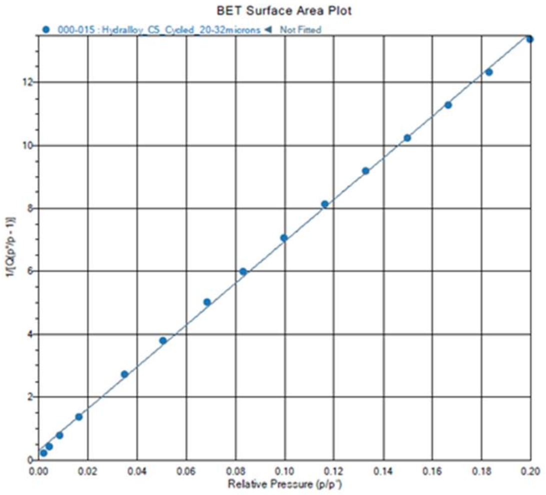

Supplementary Fig. 8: *Results of the BET surface area measurement.*

References

[1] Puszkiel JA, Neves AM, Warfsmann J, Krause PS, Kaufmann TFJ, Robelo Hoberg A, et al. On the hydrogen storage properties and life cycle evaluation of a room temperature hydride for scale-up applications: The case of an AB2-alloy. Int J Hydrogen Energy 2025;118:482–99. https://doi.org/10.1016/J.IJHYDENE.2025.03.161.

[2] Iooss B, Lemaître P. A Review on Global Sensitivity Analysis Methods. Operations Research/ Computer Science Interfaces Series 2015;59:101–22. https://doi.org/10.1007/978-1-4899-7547-8_5.

[3] Ghanem R, Higdon D, Owhadi H, editors. Handbook of uncertainty quantification. Springer International Publishing; 2017. https://doi.org/10.1007/978-3-319-12385-1.

[4] Morris MD. Factorial Sampling Plans for Preliminary Computational Experiments. Technometrics 1991;33:161. https://doi.org/10.2307/1269043.

[5] Herbrig K, Röntzsch L, Pohlmann C, Weißgärber T, Kieback B. Hydrogen storage systems based on hydride-graphite composites: Computer simulation and experimental validation. Int J Hydrogen Energy 2013;38:7026–36. https://doi.org/10.1016/j.ijhydene.2013.03.104.

[6] Passing M. Development of a thermal and hydraulic coupled hydrogen storage system based on metal hydrides for automotive application. Doctoral Thesis. Helmut-Schmidt-Universität / Universität der Bundeswehr Hamburg, 2024.
